# Ectopic Expression of *Gastrodia* Antifungal Protein in Rice Enhances Resistance to Rice Sheath Blight Disease

**DOI:** 10.3390/jof10010033

**Published:** 2023-12-31

**Authors:** Junkai Zhu, Xiang Xue, Ran Ju, Jianhua Zhao, Fen Liu, Xian Han, Yu Yan, Yu Wang, Zhiming Feng, Dongmei Lin, Zongxiang Chen, Yiqin Wang, Xijun Chen, Chengcai Chu, Shimin Zuo, Yafang Zhang

**Affiliations:** 1 Jiangsu Key Laboratory of Crop Genomics and Molecular Breeding/Zhongshan Biological Breeding Laboratory/Key Laboratory of Plant Functional Genomics of the Ministry of Education, Agricultural College of Yangzhou University, Yangzhou 225009, China; zjkzyqyt@163.com (J.Z.); yzujuran@163.com (R.J.); dx120180075@stu.yzu.edu.cn (J.Z.); lf4169531@163.com (F.L.); 17712508129@163.com (X.H.); y.0307@outlook.com (Y.Y.); dx120170066@stu.yzu.edu.cn (Y.W.); fengzm@yzu.edu.cn (Z.F.); czx@yzu.edu.cn (Z.C.); xjchen@yzu.edu.cn (X.C.); 2 Jiangsu Kingearth Seed Co., Ltd., Yangzhou 225009, China; 3 Department of Horticulture, Yangzhou Polytechnic College, Yangzhou 225009, China; 101953@yzpc.edu.cn; 4 Jiangsu Safety& Environment Technology and Equipment for Planting and Breeding Industry Engineering Research Center, Yangzhou Polytechnic College, Yangzhou 225009, China; 5 Co-Innovation Center for Modern Production Technology of Grain Crops of Jiangsu Province/Key Laboratory of Crop Genetics and Physiology of Jiangsu Province, Yangzhou University, Yangzhou 225009, China; 6 College of Horticulture and Plant Protection, Yangzhou University, Yangzhou 225009, China; m15399760504@163.com; 7 State Key Laboratory of Plant Genomics, the Innovative Academy of Seed Design, Institute of Genetics and Developmental Biology, Chinese Academy of Sciences, Beijing 100101, China; yqwang@genetics.ac.cn (Y.W.); ccchu@genetics.ac.cn (C.C.); 8 Joint International Research Laboratory of Agriculture and Agri-Product Safety, Ministry of Education of China/Institutes of Agricultural Science and Technology Development, Yangzhou University, Yangzhou 225009, China

**Keywords:** rice, sheath blight, *GAFP* genes, disease resistance, breeding value

## Abstract

Sheath blight (ShB) disease, caused by *Rhizoctonia solani* Kühn, is one of the most serious rice diseases. Rice breeding against ShB has been severely hindered because no major resistance genes or germplasms are available in rice. Here, we report that introduction of *Gastrodia* antifungal protein (GAFP) genes from *Gastrodia elata* B1 into rice significantly enhances resistance to rice ShB. Four GAFP genes were cloned from *G. elata* B1, and all displayed a strong ability to inhibit *R. solani* growth in plate assays. Two versions, with or without a signal peptide, for each of the four GAFP genes were introduced into XD3 and R6547 rice cultivars, and all transgenic lines displayed stronger ShB resistance than the corresponding wild-type control in both greenhouse and field conditions. Importantly, *GAFP2* showed the highest ShB resistance; GAFPs with and without its signal peptide showed no significant differences in enhancing ShB resistance. We also evaluated the agronomic traits of these transgenic rice and found that ectopic expression of *GAFPs* in rice at appropriate levels did not affect agronomic traits other than enhancing ShB resistance. Together, these results indicate that *GAFP* genes, especially *GAFP2*, have great potential in rice breeding against ShB disease.

## 1. Introduction

Sheath blight (ShB) disease, caused by *Rhizoctonia solani* Kühn, is a soilborne disease that is widespread among rice-growing areas in the world. It has shown a trend of becoming a more and more severe problem due to the heavy usage of nitrogen fertilizers and high-density cultivation [1]. ShB mainly causes lesions in the sheaths and leaves of plants and, thus, significantly reduces filled-grain rate, grain weight, and severely impacts rice grain yield and quality [2,3,4]. 

It is well-known that breeding disease-resistant varieties is one of the most effective and economic approaches to control ShB disease [5]. Increasing studies have shown that resistance to rice ShB is a typical quantitative trait controlled by multiple genes without major resistance genes [6,7,8,9,10]. So far, at least 50 ShB resistance quantitative loci (QTLs) have been identified with the traditional gene-mapping method, and several candidate loci or genes have been uncovered by genome-wide association analysis [11,12,13,14,15,16,17,18,19,20]. However, due to the fact that very few of these QTLs have been isolated and most of them carry only small resistance effects and their locations have not been fine-mapped, these QTLs have been seldom used in breeding practices for ShB resistance [21]. As a result, developing rice ShB-resistant varieties via marker-assisted selection for ShB QTLs has progressed very slowly.

Transgenic technology, as a promising alternative approach, has been widely used in improving rice ShB resistance by introducing either foreign genes or modified endogenous genes into rice [22,23]. For example, chitinases, which degrade chitin in fungal cell walls, enhance ShB resistance when introduced into rice [24,25]. Several studies have introduced multiple genes to improve ShB resistance. For instance, Shah et al. [26] expressed sweet protein gene *tlp-D34* and chitinase gene *chi11*, and Mao et al. [27] expressed chitinase gene *RCH10* and β-1,3-glucosidase gene *AGLU1* in rice. The results show that pyramiding multiple genes is more effective than introducing a single gene. Xue et al. [28] overexpressed the rice *OsOSM1* gene in XD3 rice, showing that it significantly enhanced ShB resistance, and found that the enhanced resistance was due to elevated jasmonate signaling. Pan et al. [29] overexpressed *JERF1* in rice and found that it enhanced ShB resistance by elevating expression of defense genes. These reports show that introducing appropriate genes into rice via the transgenic approach can improve ShB resistance, providing a feasible alternative strategy to quickly breeding ShB-resistant rice varieties [30].

*Gastrodia elata* B1, belonging to Gastrodia R. Brown of *Orchidaceae*, is an important medicinal plant with high potential. Hu et al. [31] isolated a protein from *G. elata* B1 that displayed broad-spectrum resistance to fungi and named it *Gastrodia* Antifungal Protein (GAFP). One study showed that GAFP is likely a lectin protein [32] that binds to mannose, a component present in fungal cell walls, to recognize invading fungi and exert broad spectrum resistance [33,34]. Chen et al. [35] and Cox et al. [36] separately introduced GAFP into tobacco and showed that these transgenic plants displayed resistance to *Phytophthora nicotianae* and *R. solani*. Wei et al., Chen et al., and Liu et al. [37,38,39,40] separately introduced GAFP into cotton and obtained transgenic varieties that showed enhanced resistance to cotton blight and Verticillium wilt. However, whether GAFP can enhance resistance to rice ShB disease remains unstudied.

Here, we cloned four GAFP genes from *G. elata* B1 and expressed them in rice. We found that they all enhanced resistance to ShB, with GAFP2 showing the highest level of ShB resistance. We further investigated their agronomic traits in the background of two commercial rice varieties and found no significant differences from wildtype controls, showing their application values in breeding ShB-resistant rice varieties.

## 2. Materials and Methods

### 2.1. Rice Materials

The rice varieties used for transformation were indica rice R6547 and japonica rice XD3. R6547 is a paternal donor of hybrid rice Fengyouxiangzhan and XD3 is a commercial variety widely cultivated in Jiangsu province.

### 2.2. Bioinformatic Analysis of GAFP Genes

We used the protein sequence obtained previously [41] to carry out BlastP searches (E ≤ 1 × 10^−10^) on NCBI databases and employed TBtools [42] to conduct structural validation to verify the sequences of the GAFP gene family [43]. The Neighboring-Joining method (Bootstrap replications = 1000) of the MEGA11 software was used to build the evolution tree [44]. Batch CD-search was used for conserved structural search and MEME (http://meme-suite.org/, accessed on 12 September 2023) was used for conserved amino acid sequence analysis, with settings at 8 conserved amino acids (aa), minimal 6 aa, maximal 50 aa, and the rest as default. The visual tree was built using TBtools to combine polygenetic tree, conserved amino acid sequence, and conserved structure.

### 2.3. Prokaryotic Expression and Purification of GAFPs

Sequences encoding mature peptides of GAFP1~4 were cloned into maltose-binding protein (MBP) fusion vector pETMALc-Hvia *BamH*I and *Sal*I restriction sites with C-terminal fusion to MBP [41]. In vitro expression of GAFP1~4 and purification was carried out through affinity chromatography on a column packed with amylose resins [41] as described in manufacturer’s manual. Purified proteins were digested with Thrombin to release target proteins and adjusted to a concentration approximately 2 mg.mL^−1^ for subsequent experiments.

### 2.4. Generation of GAFP Transgenic Rice Lines

GAFP1~4 in the pCAMBIA2300 vector was introduced to rice via *Agrobacterium*-mediated genetic transformation. Two versions of constructs for each GAFP peptide were introduced into rice: (1) mature peptide without the signal peptide, abbreviated as G1-G4; (2) mature peptide containing the signal peptide, abbreviated as SG1-SG4. Therefore, in total, 8 constructs were introduced into rice. The promoter of an *actin* gene was used to drive expression of these genes.

### 2.5. In Vitro Antifungal Activity Assay for GAFPs

The *R. solani* YN-7 isolate was used to evaluate the effects of GAFPs on fungal growth. A YN-7 mycelial block of 0.5 cm in diameter was placed in the center of a Petri dish and filter paper containing GAFP was placed 3 cm away. Either 10 μL, 20 μL, 30 μL, or 40 μL of GAFP was added to the filter paper. The Petri dish was kept at 28 centigrade in the dark for two days. The experiment was repeated at least twice.

### 2.6. R. solani Culture, Plant Inoculation, and Evaluation

*R. solani* YN-7, which causes mid-to-high disease severity, was maintained in our lab [45,46]. *R. solani* isolate was cultured by placing short wood sticks (10 mm × 3 mm × 0.8 mm) on a Petri dish containing sterilized PDB medium, inoculating YN-7 in the center of the Petri dish, and growing them at 28 °C in the dark for 3–5 days. The wood sticks containing mycelia were ready for plant inoculation.

Two methods of plant inoculation were used: single tiller inoculation in greenhouse and whole plant inoculation in field. Inoculation was conducted as described before [47]. Briefly, for in greenhouse inoculation, the inoculum was placed at the third sheath from top. The environmental setting was 14 h day at 30 °C/10 h night at 24 °C, with humidity at 75–90%. Lesion length was measured 14 days post inoculation. Each plant material contained 5 plants and at least three tillers for each plant were inoculated. the experiment was repeated twice.

For in-field inoculation, plants were inoculated at late tillering stage to jointing stage. Randomized field selection was used with each plant material containing three replications, with each replication containing three lines and each line containing 12 plants; the central 10 plants of the middle line were inoculated and three tillers for each plant were inoculated. Lesion length was evaluated approximately 30 days post heading using a system described before.

### 2.7. Reverse Transcription-Quantitative PCR (RT-qPCR) Analysis for Transgene Expression in Transgenic Rice

Total RNA was extracted from rice using Invitrogen TRIZOL reagent and examined on agarose gels for RNA purity and intactness. oligo(dT) was used for first-strand cDNA synthesis and the SYBR PrimeScript RT-PCR kit (TaKaRa. Dalian, China) used for RT-qPCR. *Actin* was used as the internal reference with primers: 5′-CTTCATAGGAATGGAAGCTGCGGGTA-3′ and 5′-CGACCACCTTGATCTTCATGCTGCTA-3′. Primers for the 4 *GAFP* genes were 5′-GCCGTAAAGACTGGCGAACA-3′ and 5′-GGAAGGGTCTTGCGAAGGAT-3′. Three biological replications were included for each sample.

### 2.8. Western Blotting Analysis for Transgene Expression in Transgenic Rice

Total proteins were extracted from *GAFP* transgenic rice leaves at the 4-leaf developing stage using protein extraction buffer (50 mM HEPES [pH7.5], 150 mM KCl, 1 mM DTT, 0.4% Triton-X 100, and proteinase inhibitor cocktail). Then, the proteins were separated on SDS-PAGE gels and immunoblotted with a GAFP polyclonal antibody developed by Wang et al. (2016), previously used to detect GAFP proteins [41]. HSP82 antibody (Beijing Protein Innovation. Beijing, China) was used to detect endogenous HSP82 protein, which was used as the loading control.

### 2.9. Evaluation of Agronomic Traits

Major agronomic traits of R6547, XD3, and transgenic lines were evaluated. Evaluated agronomic traits include heading date, plant height, panicle length, flag leaf length and width, number of panicles with filled grains, filled grains per panicle, filled grain rate, 1000-grain weight, grain weight per plant, grain length and width, chalky grain rate, chalkiness degree, and amylose content (AC). Each rice material contained 3 replicate areas, each area containing 3 lines and each line containing 12 plants. The 10 plants in the middle of each area were used for measurement. Measurement was conducted as described by He et al. [47]. Three replications were included to calculate for the mean value.

### 2.10. Data Analysis

Data of sheath blight disease severity and agronomic traits were organized in Excel 2016. Significant differences between different transgenic lines and the corresponding control variety were analyzed using Student’s *t*-test in the SPSS 24.0 program.

## 3. Results

### 3.1. GAFP Genes Are Mainly Present in Monocotyledonous Plants of the Orchidaceae Family

**Figure 1:**
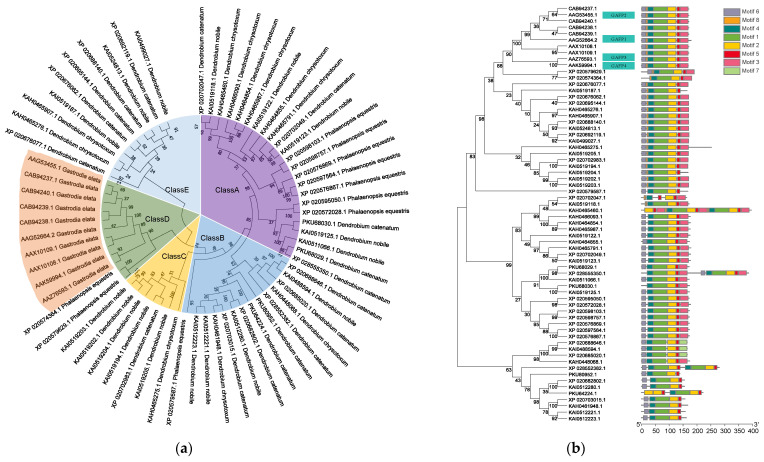
Bioinformatic analysis of *GAFP* genes. (**a**) Evolution tree of *GAFP* genes of the Orchidaceae family. The genes are grouped into five classes: A–E; (**b**) evolution tree of *GAFP* genes and their conserved motifs. *Gastrodia elata* GAFP1, GAFP2, GAFP3, and GAFP4 share 85% similarity.

Via analyzing NCBI databases, we found that *GAFP* genes are mainly present in monocotyledonous plants of the *Orchidaceae* family, including *G. elata*, *Phalaenopsis equestris*, and *Dendrobium nobile*, but not in common model plants *Arabidopsis*, rice, or tobacco. BlastP analysis revealed 10, 10, and 46 *GAFP* homologous genes for *G. elata*, *P. equestris*, and the *Dendrobium genus*, respectively. *Dendrobium*, *D*. *catenatum*, *D. chrysotoxum*, and *D. nobile* contain 18, 11, and 17 *GAFP* homologous genes, respectively (Supplementary Table S1). We built an evolution tree for the 66 *GAFP* genes using MEGA11 (Figure 1a) and found that they could be divided into five (A-E) classes: Class D contains most *G. elata* GAFPs (ten genes) and two *P. equestris* GAFPs, suggesting that *G. elata* and *P. equestris* GAFPs are more evolutionarily related. The other *P. equestris* GAFPs are mostly located in Class A (seven genes) and less in Class B (one gene). *Dendrobium* GAFPs are distributed in Classes A, B, C, and E, but not in Class D. Classes C and E contain only *Dendrobium* GAFPs. These results suggest that *Dendrobium* GAFP genes are more distantly related to *G. elata* and *P. equestris ones*.

The Batch CD-search revealed that all GAFP members contain a conserved B-lectin motif and at least a motif 3, a motif 4, and a motif 5 (Figure 1b, Supplementary Table S2). Among the *G. elata* GAFPs, four of them share over 85% similarity: GAFP1 (AAG52664.2), GAFP2 (AAG53455.1), GAFP3 (AAK59994.1), and GAFP4 (AAZ76593.1). Normally, *G. elata* can be divided into four different varieties: *G. elata* Bl. *F. elata*, *G. elata* Bl. *F. viridls* MalKino, *G. elata* Bl. *F. glauca* S Chow, and *G. elata* Bl. *F. flavida* S Chow. The four GAFPs with the highest similarity, which were selected for further experiment, are all from variety *G. elata* B1. *F elata*.

### 3.2. GAFPs Proteins Inhibit R. solani Growth on Plates

**Figure 2:**
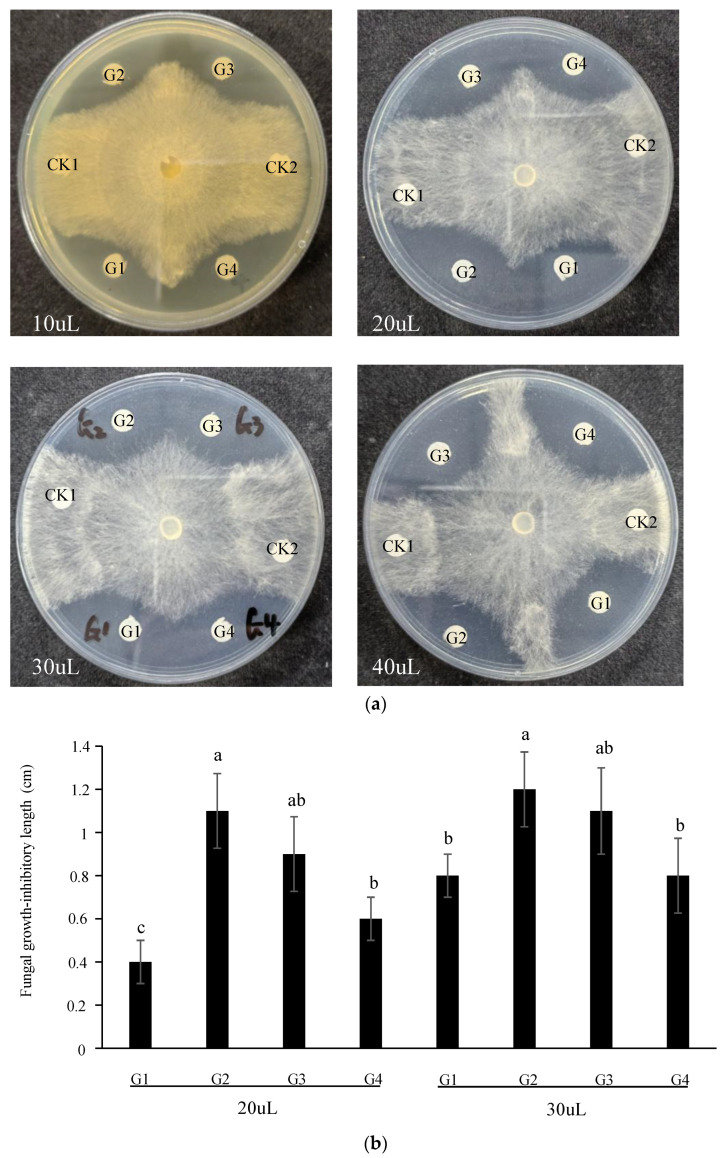
Fungal growth inhibitory assay for GAFP proteins. (**a**) G1-G4 denote inclusion of GAFP1-GAFP4 proteins; CK1 denotes water control and CK2 denotes BSA. GAFP proteins were added in 10 μL, 20 μL, 30 μL, and 40 μL amounts in different plates. (**b**) Activity of GAFP proteins in inhibiting *R. solani* growth in plate assays. The inhibition distances of GAFP proteins were measured from the site of protein to the closest *R. solani* hyphae on plate. Because no clear differences between different GAFP proteins on inhibiting *R. solani* growth in treatments with 10 μL and 40 μL were found, these treatments were therefore not included in this figure. Different lowercase letters on the bar represent the significant difference at 5% statistical significance level.

We cloned the four *G. elata GAFP* (*GAFP1-4*) genes that are highly homologous to evaluate their effects on *R. solani* growth on plates. We expressed these four genes in *E. coli*, purified them, and evaluated their effects on *R. solani* mycelial growth in cultural medium (Figure 2a). We found that all four GAFP proteins significantly inhibited *R. solani* mycelial growth at all four concentrations. We further measured the distances (mm) between the GAFP proteins and the closest *R. solani* mycelia for treatments with 20 μL and 30 μL protein; almost no visible differences were observed for the 10 μL and 40 μL treatments. We found that the distances of G2 and G3 were significantly longer than those of G1 and G4 in both treatments (Figure 2b), suggesting that G2 and G3 are more effective in inhibiting *R. solani* mycelial growth. No significant differences between G2 and G3 in inhibiting *R. solani* hypha growth were found, although the average distance of G2 was longer than that of G3. Together, these data showed that G2 and G3 were slightly more effective in inhibiting *R. solani* growth than G1 and G4 in the 20 and 30 μL treatments.

### 3.3. Transgenic GAFPs Rice Plants Were Generated and Verified

**Figure 3:**
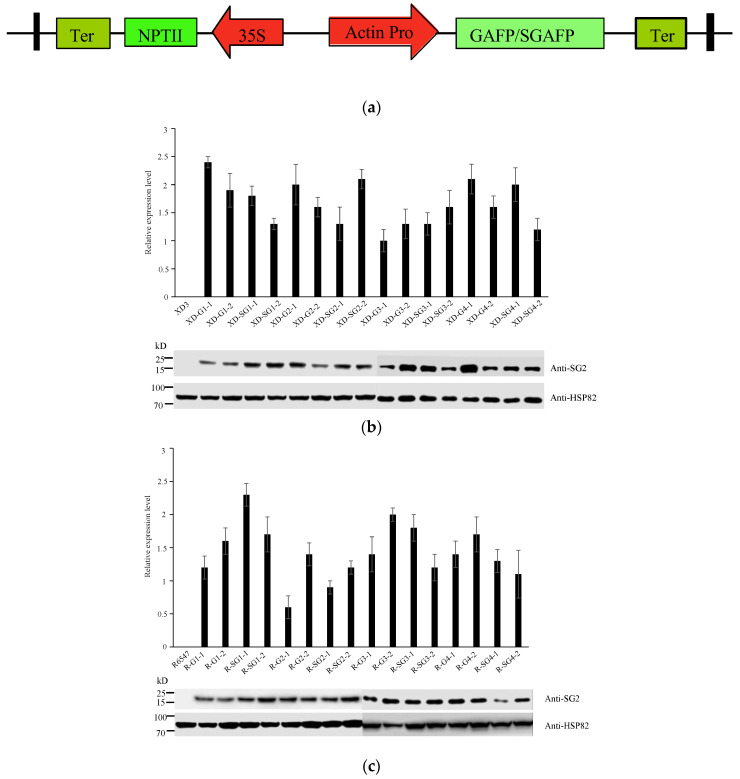
Characterization of GAFP transgenic lines.(**a**) Partial map of GAFP constructs. (**b**,**c**) GAFP RNA and protein levels in XD3 and transgenic lines (**b**) or in R6547 and transgenic lines (**c**) detected by RT-qPCR and immunoblotting, respectively.

In order to test whether these GAFPs can confer ShB resistance in rice, we made eight constructs, in which an actin promoter (Actin pro) drives expression of G1-G4 and SG1-SG4 DNA sequences individually (Figure 3a) and generated transgenic rice expressing each of these constructs in two rice varieties. We obtained 4–9 independent lines for each construct in each genetic background and verified them by PCR. We then selected two lines for each that showed similar agronomic traits to their wildtype control and analyzed their transgene expression levels by RT-qPCR and immunoblotting analyses. The results showed that while the two wildtype controls expressed no *GAFP* RNA, all GAFPs’ transgenic lines expressed significant levels of GAFP RNA. All GAFPs’ transgenic lines also displayed a clear band at 15KD, as detected by an antibody against G2 protein. We also observed that GAFP protein levels showed higher variations in the XD3 genetic background than those in the R6547 genetic background (Figure 3b,c).

### 3.4. Transgenic GAFPs Rice Lines Display Enhanced Resistance to R. solani

**Figure 4:**
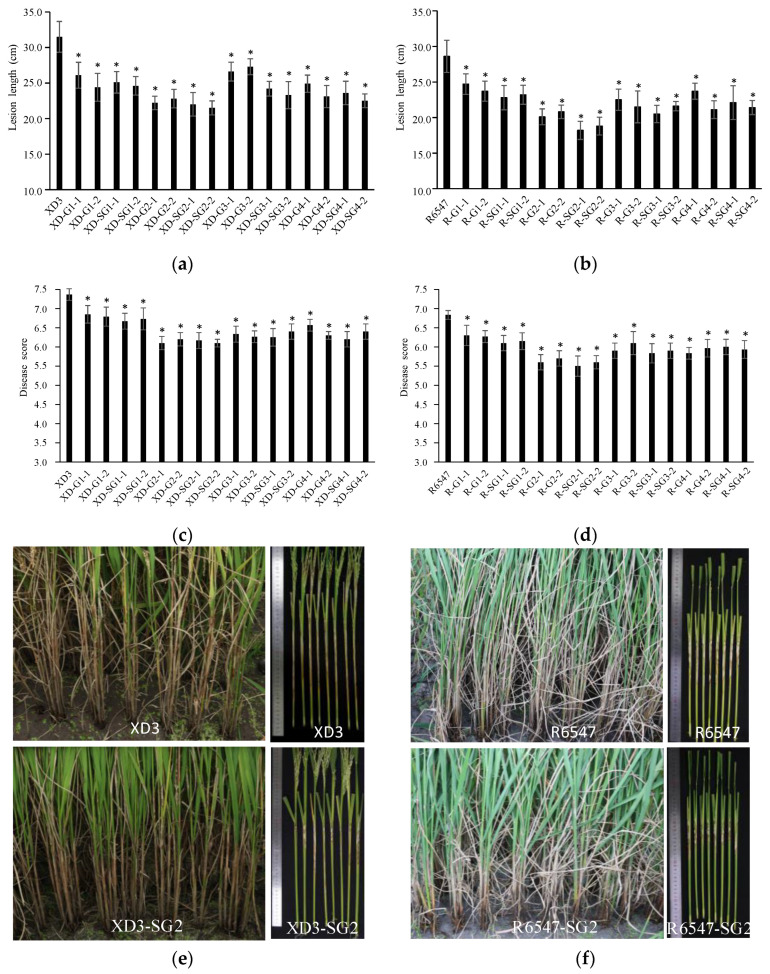
ShB resistance levels of different GAFP transgenic lines and their controls. (**a**,**b**) ShB lesion lengths of XD3 and transgenic lines (**a**) or R6547 and transgenic lines (**b**) in greenhouse inoculation assay. (**c**,**d**) ShB disease scores of XD3 and transgenic lines (**c**) or R6547 and transgenic lines (**d**) in field inoculation test. E–F. ShB disease pictures of XD3 and transgenic lines (**e**) or R6547 and transgenic lines (**f**) in greenhouse assay or in field inoculation test. * Indicates 5% level significant differences.

We first evaluated ShB resistance for these GAFPs transgenic lines in the greenhouse. We found significantly shorter lesion lengths in all transgenic lines compared to the corresponding wild-type control plants in both the XD3 and R6547 genetic background (Figure 4a,b). In the XD3 background, while XD3 developed an average lesion length of 31.5 ± 2.17 cm, GAFPs’ transgenic lines showed lesion lengths of 21.5 ± 0.98~27.3 ± 1.11 cm (Figure 4a,e). G2 and SG2 lines showed the shortest lesion lengths (21.5 ± 0.98~22.8 ± 1.30 cm) among all lines. In addition, G2 and SG2 were not significantly different from each other. The G1 and SG1 lines displayed the longest lesion lengths (averaged 27.3 ± 1.11 cm) among the GAFPs lines. In the R6547 background, while R6547 developed an average lesion length of 28.6 ± 2.26 cm, GAFPs’ lines developed average lesion lengths of 18.2 ± 1.28 cm~24.7 ± 1.45 cm, all significantly shorter than the control. Similarly, the G2 and SG2 lines showed the shortest lesions (Figure 4b,f), especially the two SG2 lines displaying lesion lengths of 18.2 ± 1.28 cm and 18.8 ± 1.25 cm, being 10.1 cm shorter than the control. G1 and SG1 also had the weakest resistance-enhancing effects.

We further evaluated their effects in a field test and found that the disease severity scores of these transgenic lines were also significantly lower than those of the controls (Figure 4c,d). In the XD3 background, while XD3 scored 7.37 ± 0.15, GAFPs’ transgenic lines scored 6.10 ± 0.10~6.85 ± 0.23, being 0.52~1.27 less severe than the control (Figure 4c,e). G2 and SG2 were the most resistant lines, with scores of 6.10 ± 0.10~6.20 ± 0.17 cm, being 1.2 lower than the control. G1 and SG1 showed the lowest enhanced resistance among GAFPs’ transgenic lines. In the R6547 background, while the R6547 control scored 6.83 ± 0.12, GAFPs’ transgenic lines scored 5.50 ± 0.26~6.30 ± 0.26, being 0.53~1.33 lower than the control (Figure 4d,f). Similarly, G2 and SG2 showed the highest resistance and G1 and SG1 showed the lowest enhanced resistance. These results suggest that GAFPs confer enhanced resistance to ShB in rice and G2 has the most significant effects.

**Figure 5:**
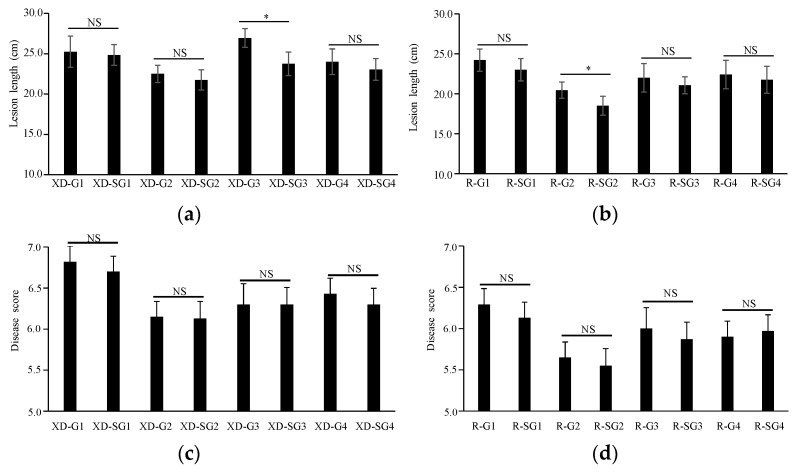
Effects of GAFP proteins with or without a signal peptide on ShB resistance. (**a**,**b**) Lesion lengths of lines expressing GAFPs with or without a signal peptide in XD3 (**a**) or R6547 (**b**) background after *R. solani* inoculation in greenhouse. (**c**,**d**). Disease scores of lines expressing GAFPs with or without a signal peptide in XD3 (**c**) or R6547 (**d**) background after *R. solani* inoculation at late tillering stage in a field test. * Indicates 5% level significant differences. NS indicates no significant difference.

We further assessed the function of the signal peptide of GAFPs in disease resistance and found no significant differences between lines with or without the signal peptide, except for G2 vs. SG2 in the XD3 background and G3 vs. SG3 in the R6547 background in the greenhouse assay (Figure 5a,b). Because in both significant cases the SG lines developed shorter lesions than the G lines and other SG lines tended to have shorter lesions than their corresponding G lines (Figure 5a–d), GAFP constructs carrying a signal peptide may be more stable in conferring resistance to ShB in rice.

### 3.5. Most GAFP Transgenic Lines Display No Significant Differences from Wild-Type Controls in Major Agronomic Traits

**Figure 6:**
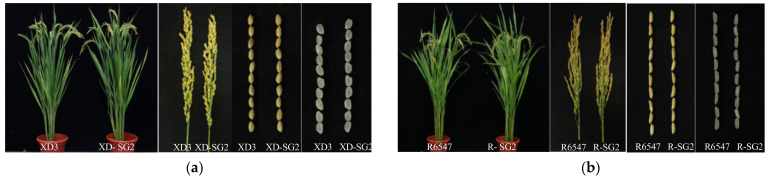
Comparison of whole plants, panicles, and grains of GAFP transgenic lines and controls. (**a**) Whole plants, panicles, and grains of XD3 and transgenic line XD-SG2. (**b**) Whole plants, panicles, and grains of R6547 and transgenic line R-SG2.

To further evaluate the application potential of *GAFP* genes in ShB resistance, we compared major agronomic traits, including grain yield, of the transgenic lines with those of the wild-type controls (Table 1, Table 2, Table 3 and Table 4, Figure 6). We found no consistent trend of deviation in the transgenic plants compared to the wild-type controls, despite some sporadic variations (Table 1 and Table 3). For example, XD-SG1 and XD-SG3, but not other lines in the XD3 background, displayed shorter heading dates. XD-SG2 showed a shorter plant height but XD-G4 showed a longer plant height than wild-type XD3. For grain yield, only the line XD-G4, but not other lines, showed a significantly lower yield than the wild-type XD3. Thus overall, no consistently significant agronomic trait changes in the transgenic lines were found.

Similar results were observed for GAFPs’ transgenic lines in the R6547 background (Table 2 and Table 4). Some sporadic variations were found but no consistent deviating trends were observed for the transgenic lines, especially for grain yield. These variations were not concentrated on common traits, which allowed us to reason that these sporadic variations were most likely caused by somatic mutations in the tissue culture process but not by *GAFP* gene introduction. We therefore conclude that via agronomic selection first, the introduction of GAFP genes into rice does not cause significant agronomic changes.

### 3.6. Inappropriately High Levels of GAFP Expression May Result in Shorter Plants and Other Agronomic Trait Changes

**Figure 7:**
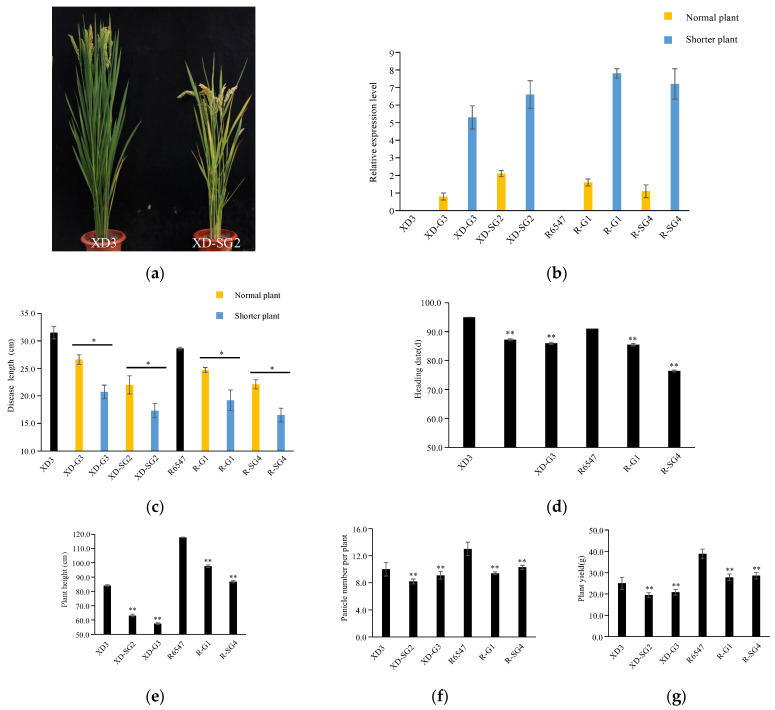
Comparison of plant morphology, ShB resistance, and agronomic traits of normal and shorter transgenic plants. (**a**) Plant morphology of XD3 and transgenic line XD-SG2. (**b**) Comparison of GAFP RNA levels of two different transgenic lines with normal or shorter plant height for XD-G3, XD-SG2, R-G1, and R-SG4. (**c**–**g**) ShB lesion length (**c**), heading date (**d**), plant height (**e**), panicle numbers per plant (**f**), and grain yield per plant (**g**) of transgenic lines with normal or shorter height and controls. * Indicates 5% and ** indicates 1% level significant differences.

Despite the fact that no consistent overall trends in agronomic trait changes were found for GAFPs’ transgenic lines, we further investigated the potential correlation between deviating phenotypes and *GAFPs’* expression levels. We first observed that some independent T0 lines carrying the same GAFP construct displayed shorter plant heights than other independent lines and monitored their *GAFP* expression levels. We found that these shorter plants expressed particularly higher *GAFP* RNA levels (over 5-fold of actin RNA level) than other lines (about 0.5~2-fold of actin RNA level) carrying the same construct (Figure 7a,b). This variation was observed for some lines of XD-SG2, XD-G3, R-G1, and R-SG4. We then measured their ShB resistance and agronomic traits and found that their higher *GAFP* RNA levels were correlated with higher ShB resistance, shorter heading dates, shorter plant heights, lower panicle numbers per plant, and lower grain yields (Figure 7c–g).

These results indicate that inappropriately high levels of *GAFP* expression in rice may result in undesirable agronomic trait changes, leading to yield loss. However, since most transgenic lines did not have their plant heights affected, this defect can be easily avoided by choosing T0 lines that show normal plant heights.

## 4. Discussion

### 4.1. Different GAFP Genes May Have Different Effects against Different Pathogens

We found that GAFP genes are mainly present in the *Orchidaceae* family, including *G. elata* (Figure 1a), but not in the *Poaceae* (*Gramineae*) family. Previously, we tested the four *GAFP* genes against the growth of pathogens *Trichoderma viride* and *Verticillium dahliae* and found that G4 has the highest activity against these two pathogens [41]. However, in this report, we found that G2 has the highest activity against *R. solani* growth, followed by G3 and G4, then G1 (Figure 2). In consistency, G2 also conferred the highest level of ShB resistance in transgenic rice lines (Figure 4). These results clearly indicate that different *GAFP* genes have different levels of effects on different pathogens. So far, the mechanism underling this difference remains unclear. Because GAFPs are mannose-binding lectins, the different GAFP antifungal activities may be caused by the different compositions in fungal cell walls. However, the correlation between different GAFP proteins and their activities against different pathogens still needs further study. Thus, for an untested pathogen, one would have to test different *GAFP* genes for the best result.

The GAFP family contains sequences encoding a 28 aa signal peptide at their N termini, and our previous study showed that the signal peptide ensures secretion of GAFPs to the apoplast [43]. In the test against cotton blight and cyanosis disease, SG4 showed better effects than G4, suggesting that inclusion of the signal peptide in the G4 construct may have helped secretion of the protein and facilitated its activity against the pathogen outside the host cell [41]. In the present study, we only observed two cases (SG3 in XD3 and SG2 in R6547) where the presence of a signal peptide resulted in significant differences in ShB resistance (Figure 5). However, considering that the overall trend shows that GAF constructs carrying a signal peptide have better results in enhancing ShB resistance than those that do not carry a signal peptide, we suggest that a signal peptide be included when making a GAFP construct to be introduced into plants.

### 4.2. GAFP Genes Carry High Values in the Breeding against Rice ShB Disease

Liu et al. [48] evaluated the effects of disease resistance on agronomic traits and economic value upon introduction of the *G1* gene into color cotton and found that the resistance against cotton blight and cyanosis disease conferred by GAFP significantly impacted agronomic traits and economic value. Shen et al. [49] introduced a *GAFP* gene into Xinjiang upland cotton and found that the transgenic lines not only displayed higher resistance to cotton blight and cyanosis disease, but also higher economic value and better agronomic traits. Here, with the aim for application, we first selected GAFP transgenic lines that showed agronomic traits indistinguishable from those of the wild-type controls, and then we used these lines for further characterization, including ShB resistance assay. This approach avoided the downside of selecting lines with high ShB resistance but carrying undesirable agronomic traits. We found that the *GAFP* RNA expression levels of our selected lines were 0.5~2.5-fold of the actin RNA level (Figure 3b,c). This approach clearly avoided transgenic lines that expressed inappropriately high levels of *GAFP* RNA.

On the other hand, we also investigated several lines that displayed shorter plant heights and found their *GAFP* RNA expression levels were higher than those with normal heights. For example, we found four transgenic lines that showed shorter heading dates and this phenotype was linked to lower yield (Figure 7). Further RT-qPCR results showed that they expressed much higher *GAFP* RNA levels, at least 5-fold of the *actin* RNA level. These results strongly indicate that although high levels of GAFPs strongly enhance ShB resistance, they can also detrimentally affect agronomic traits and are therefore undesirable. Given the above-described bottom lines, we conclude that SG2 carries a high value for breeding ShB-resistant rice varieties. In future rice breeding against ShB disease, one needs to further increase the number of *SG2* transgenic lines to obtain lines with optimal expression levels that acquire the highest ShB resistance with no inferior effects on yield.

With regard to protein levels, we found some variabilities in different XD3 transgenic lines, although their transcription levels had no significant difference. In Figure 3b, the four transgenes each carry a pair of lines with variable protein levels compared to each other. For example, the line XD-G2-1 carries a protein level higher than the line XD-G2-2, the line XD-G3-2is higher than the line XD-G3-1, the line XD-SG3-1 is higher than XD-SG3-2, and the line XD-G4-1 is higher than the line XD-G4-2. However, in each pair, both transgenic lines showed similar resistance with no significant differences in greenhouse or field condition (Figure 4a,c). With regard to different GAFP genes, we found that XD-G1-1 and XD-G1-2 showed similar protein levels with XD-G2-2, but XD-G2-2 had significantly stronger resistance than XD-G1-1 and XD-G1-2; XD-SG1-1 and XD-SG1-2 showed similar levels with XD-G2-1, but XD-G2-1 showed stronger resistance than XD-SG1-1 and XD-SG1-2. Some G3 and G4 lines showed higher protein levels than the G2 lines but lower resistance than the G2 lines. Moreover, in the R6547 background, most transgenic lines showed similar protein expression levels and resistance. Therefore, we believe that although variability occurs in GAFP protein levels in XD3 transgenic lines, this variability does not affect our conclusion or rice development.

## Figures and Tables

**Table 1:** Major agronomic traits and economic value evaluation of XD3 and transgenic lines.

Line	Heading Date (d)	Plant Height (cm)	Panicle Length (cm)	Flag Leaf Length (cm)	Flag Leaf Width (cm)	Panicle Number per Plant	Grain Number per Panicle	Filled Grain Rate (%)	1000-Grain Weight (g)	Grain Yield per Plant (g)
XD3	95.0 ± 0.00	84.1 ± 0.50	18.9 ± 0.26	23.8 ± 0.72	1.5 ± 0.17	10.0 ± 1.00	105.3 ± 3.51	91.8 ± 1.75	26.8 ± 0.36	25.0 ± 2.80
XD-G1	94.0 ± 0.89	83.9 ± 1.59	18.5 ± 0.58	23.4 ± 0.87	2.4 ± 0.20 *	11.0 ± 2.00	110.0 ± 7.04	93.4 ± 0.26 *	26.2 ± 0.50	27.7 ± 4.91
XD-SG1	92.5 ± 0.00 *	83.3 ± 0.42	19.1 ± 0.69	19.4 ± 1.06 **	1.5 ± 0.18	10.5 ± 1.05	104.5 ± 9.14	92.3 ± 1.51	27.1 ± 0.38	25.9 ± 2.43
XD-G2	94.2 ± 0.41	84.9 ± 0.98	22.5 ± 0.40 *	23.3 ± 0.72	1.5 ± 0.17	9.5 ± 1.52	105.3 ± 5.96	92.2 ± 0.46	26.8 ± 0.40	24.0 ± 4.47
XD-SG2	94.2 ± 0.41	81.1 ± 0.92 *	19.0 ± 0.81	24.0 ± 0.79	1.4 ± 0.13	9.0 ± 1.41	107.5 ± 7.01	90.8 ± 1.45	26.7 ± 0.51	22.4 ± 3.85
XD-G3	95.0 ± 0.63	84.4 ± 1.04	19.1 ± 0.36	23.6 ± 0.98	2.6 ± 0.13*	10.5 ± 1.05	112.5 ± 3.64 *	92.6 ± 1.33 *	26.7 ± 0.41	25.3 ± 3.12
XD-SG3	92.5 ± 0.63 *	83.0 ± 0.83	23.5 ± 0.99 **	23.3 ± 0.74	1.6 ± 0.23	9.5 ± 1.05	102.5 ± 6.80	91.2 ± 1.62	26.5 ± 0.35	22.7 ± 3.43
XD-G4	94.0 ± 0.00	88.0 ± 1.00 **	19.2 ± 0.46	24.8 ± 0.66	1.5 ± 0.14	8.5 ± 1.05	103.0 ± 4.10	91.5 ± 1.98	24.6 ± 0.51 *	21.2 ± 1.69 **
XD-SG4	94.0 ± 0.63	84.4 ± 1.14	15.4 ± 0.83 *	26.3 ± 0.63 **	1.5 ± 0.24	9.7 ± 1.21	110.0 ± 8.10	92.6 ± 1.38	27.4 ± 0.26	22.3 ± 2.07

* Indicates 5% and ** indicates 1% level significant differences.

**Table 2:** Major agronomic traits and economic value evaluation of R6547 and transgenic lines.

Line	Heading Date (d)	Plant Height (cm)	Panicle Length (cm)	Flag Leaf Length (cm)	Flag Leaf Width (cm)	Panicle Number per Plant	Grain Number per Panicle	Filled Grain Rate (%)	1000-Grain Weight (g)	Grain Yield per Plant (g)
R6547	91.0 ± 0.00	117.7 ± 0.30	23.6 ± 0.46	32.0 ± 0.40	2.3 ± 0.25	13.0 ± 1.00	158.0 ± 4.36	85.5 ± 3.46	24.8 ± 0.30	38.8 ± 2.25
R-G1	90.5 ± 0.84	117.3 ± 1.42	24.5 ± 0.55	33.9 ± 0.70	2.6 ± 0.18 *	13.0 ± 1.41	160.0 ± 6.26	85.8 ± 1.16	25.2 ± 0.37	39.0 ± 3.96
R-SG1	91.0 ± 0.23	116.0 ± 1.73	21.3 ± 0.74 *	33.8 ± 2.45	2.5 ± 0.22	11.5 ± 1.05	159.0 ± 6.99	86.1 ± 1.28	24.4 ± 0.63	36.8 ± 3.71
R-G2	89.0 ± 0.34 *	117.1 ± 0.63	23.2 ± 0.43	29.0 ± 0.24 *	2.5 ± 0.19	14.3 ± 1.37	159.0 ± 2.61	85.8 ± 0.79	25.0 ± 0.35	39.7 ± 3.12
R-SG2	90.5 ± 0.55	117.4 ± 0.94	23.2 ± 0.39	35.1 ± 1.61 *	2.4 ± 0.32	13.5 ± 1.38	157.0 ± 6.63	84.8 ± 1.70	24.7 ± 0.53	38.5 ± 3.84
R-G3	91.0 ± 0.63	113.6 ± 0.85 *	21.2 ± 0.23 *	31.3 ± 1.59	2.3 ± 0.17	14.0 ± 1.79	156.0 ± 8.72	86.8 ± 1.16	25.4 ± 0.63	40.6 ± 3.99
R-SG3	88 ± 0.55 **	118.7 ± 1.68	24.8 ± 2.03	34.4 ± 2.31	2.4 ± 0.10	12.5 ± 1.87	157.5 ± 8.24	83.2 ± 1.87 *	24.9 ± 0.24	36.9 ± 4.20
R-G4	90.5 ± 0.84	119.3 ± 1.28	24.5 ± 0.63	32.7 ± 0.61	2.4 ± 0.21	13.5 ± 1.05	155.0 ± 5.97	87.1 ± 1.08	24.4 ± 0.50	38.7 ± 1.80
R-SG4	92.0 ± 0.00	122.8 ± 0.59 **	23.8 ± 0.68	29.8 ± 0.70	2.1 ± 0.24	15.0 ± 1.55	156.0 ± 4.10	87.8 ± 0.24	25.8 ± 0.21 *	39.6 ± 0.26

* Indicates 5% and ** indicates 1% level significant differences.

**Table 3:** Major grain quality traits of XD3 and transgenic lines.

Line	Grain Length (cm)	Grain Width (cm)	Chalkiness Rate (%)	Chalkiness Degree (%)	AC (%)
XD3	0.70 ± 0.030	0.36 ± 0.026	18.7 ± 0.50	3.7 ± 0.36	18.4 ± 0.75
XD-G1	0.71 ± 0.023	0.35 ± 0.017	17.3 ± 0.24 **	3.8 ± 0.36	18.0 ± 0.73
XD-SG1	0.73 ± 0.031	0.36 ± 0.024	16.9 ± 0.59 **	3.6 ± 0.24	18.0 ± 0.43
XD-G2	0.69 ± 0.030	0.35 ± 0.020	18.8 ± 0.62	3.8 ± 0.62	16.6 ± 0.55 **
XD-SG2	0.69 ± 0.024	0.35 ± 0.030	18.3 ± 0.24	3.6 ± 0.62	18.0 ± 0.74
XD-G3	0.70 ± 0.015	0.36 ± 0.014	18.9 ± 0.58	3.1 ± 0.32	17.5 ± 0.25 *
XD-SG3	0.73 ± 0.028	0.34 ± 0.014	18.8 ± 0.37	5.9 ± 0.74 *	18.3 ± 0.73
XD-G4	0.62 ± 0.014 *	0.35 ± 0.010	18.6 ± 0.32	3.6 ± 0.91	18.5 ± 0.49
XD-SG4	0.73 ± 0.026	0.34 ± 0.020	18.8 ± 0.78	3.6 ± 0.50	18.1 ± 0.64

* Indicates 5% and ** indicates 1% level significant differences.

**Table 4:** Major grain quality traits of R6547 and transgenic lines.

Line	Grain Length (cm)	Grain Width (cm)	Chalkiness Rate (%)	Chalkiness Degree (%)	AC (%)
R6547	0.89 ± 0.030	0.24 ± 0.026	16.5 ± 0.75	1.8 ± 0.35	14.2 ± 0.35
R-G1	0.83 ± 0.020 *	0.24 ± 0.010	14.8 ± 0.37 *	1.9 ± 0.55	13.3 ± 0.51
R-SG1	0.89 ± 0.028	0.24 ± 0.026	16.4 ± 0.64	1.7 ± 0.36	14.0 ± 0.64
R-G2	0.92 ± 0.024	0.23 ± 0.014	16.5 ± 0.42	2.7 ± 0.27 **	14.0 ± 0.40
R-SG2	0.91 ± 0.023	0.23 ± 0.015	16.9 ± 0.37	1.9 ± 0.32	13.8 ± 0.24
R-G3	0.90 ± 0.028	0.24 ± 0.014	17.9 ± 0.28 *	2.0 ± 0.45	14.4 ± 0.53
R-SG3	0.82 ± 0.016 *	0.22 ± 0.014	16.9 ± 1.47	2.8 ± 0.34 **	13.9 ± 0.82
R-G4	0.91 ± 0.024	0.24 ± 0.009	16.6 ± 1.04	1.7 ± 0.31	13.6 ± 0.68
R-SG4	0.89 ± 0.032	0.23 ± 0.015	16.7 ± 1.18	1.6 ± 0.24	13.8 ± 0.24

* and ** indicate 5% and 1% level significant differences, respectively.

## Data Availability

Data are contained within the article and supplementary materials.

## Supplementary Materials

The following supporting information can be downloaded at: https://www.mdpi.com/article/10.3390/jof10010033/s1, Table S1: *Gastrodia elata*, *Phalaenopsis equestris*, and *Dendrobium* GAFP proteins sequences ; Table S2. Sequences of conserved motifs in GAFPs.
