# Supplementary material for: Ectopic Expression of Gastrodia Antifungal Protein in Rice Enhances Resistance to Rice Sheath Blight Disease
Source: J Fungi (Basel). 2023 Dec 31;10(1):33. doi: 10.3390/jof10010033 (PMC10820164; doi:10.3390/jof10010033)
Supplement: Supplementary file 1 [file jof-10-00033-s001.zip › Supplementary Tables.pdf]

Supplementary Table S1. *Gastrodia elata*, *Phalaenopsis equestris*, and *Dendrobium* GAFP proteins sequences

Protein id	Organism	Protein sequence
AAZ76593.1	*Gastrodia elata*	MASPASSAVIFFFAVAALMSLLAMPALAASQLNAGQTLGTGQSLAQGPNQFIIQNDCNLVLYASNKAVWATGTNGKASGCVLRMQRDGNLVIYSGSKV<br>IWASNTNRRDDNYYLLLQRDRNVVIYDSSNNAIWSTGTNLGNAAVTVIPHSNGTAAASGAAQNKVNEYLRP
AAK59994.1	*Gastrodia elata*	MASPASSAVIFLFAVAALMSLLAMPALAASQLNAGQTLGTGQSLAQGPDQFVIQNDCNLVLYDSNRVVWASGTNGKASGCVLRMQRDGNLVIYSGSR<br>VIWASNTNRRDDNYYLLLQRDRNVVIYDSSNNAIWATGTNVGNAAITVIPHSNGTAAASGAAQNKVNEYLRP
AAX10108.1	*Gastrodia elata*	MAASASTAVILFFAVTTMMSLSAIPAFASDRLNAGKSLGAGGSLAQGPYLFIMQSDCNLVLYDNNRAVWASGTNGKASNCILKMQRDGNLVIYSGSRA<br>MWASNTNRQDGNYYLILQRDRNVVIYDNSNNAIWASGTNVGNAEITVIAHSNGTAAASGAAQNKVNELYISMY
AAX10109.1	*Gastrodia elata*	MAASASTAVILFFAVTTMMSLSAIPAFASDRLNAGKSLNTGASLAQGGYLFIMQSDCNLVLYDNNKAIWASGTNGKASNCILKMQRDGNLVIYSGSRAI<br>WASNTNRQDGNYYLILQRDRNVVIYDNSNNAIWASGTNVGNAEITVIAHSNGTAAASGAAQNKVNELYISMY
CAB94238.1	*Gastrodia elata*	MAASASTAVILFFAVTTMMSLSAIPAFASDRLNSGHQLDTGGSLAQGGYLFIIQNDCNLVLYDNNRAVWASGTNGKASGCMLKMQNDGNLVIYSGSRA<br>IWASNTNRQNGNYYLILQRDRNVVIYDNSNNAIWATHTNVGNAEITVIPHSNGTAAASGAAQNKVNELYISMY
CAB94239.1	*Gastrodia elata*	MAASASTAVILFFAVTTVMSLSAIPAFASDRLNSGHQLDTGGSLAQGGYLFIIQNDCNLVLYDNNRAVWASGTNGKASGCMLKMQNDGNLVIYSGSRAI<br>WASNTNRQNGNYYLILQRDRNVVIYDNSNNAIWATHTNVGNAEITVIPHSNGTAAASGAAQNKVNELYISMY
AAG52664.2	*Gastrodia elata*	MAASASTAVILFFAVTTMMSLSAIPAFASDRLNSGHQLDTGGSLAQGGYLFIIQNDCNLVLYDNNRAVWASGTNGKASGCVLKMQNDGNLVIYSGSRAI<br>WASNTNRQNGNYYLILQRDRNVVIYDNSNNAIWATHTNVGNAEITAIPHSNGTAAASGAAQNKVNELYISMYSRSKRIAG
CAB94240.1	*Gastrodia elata*	MAASASTAVILFFAVTTVMSLSAIPAFASDRLNSGHQLDTGGSLAQGGYLFIIQNDCNLVLYDNNRAVWASGTNGKASNCFLKMQNDGNLVIYSGSRAI<br>WASNTNRQNGNYYLILQRDRNVVIYDNSNNAIWATHTNVGNAEITVIPHSNGTAAASGAAQNKVNELYISMY
CAB94237.1	*Gastrodia elata*	MAASASTAVILFFAVTTMMSLSAIPAFASDRLNSGHQLDTGGSLAQGGYLFIIQNDCNLVLYDNNRAVWASGTNGKASNCFLKMQNDGNLVIYSGSRAI<br>WASNTNRQNGNYYLILQRDRNVVIYDNSNNAIWATHTNVGNAEITVIPHSNGTAAASGAAQNKVNELYISMY
AAG53455.1	*Gastrodia elata*	MAASASTAVILFFAVTTMMSLSAIPAFASDRLNSDHQLDTGGSLAQGGYLFIIQNDCNLVLYDNNRAVWASGTNGKASNCFLKMQNDGNLVIYSGSRAI<br>WASNTNRQKGNYYLILQRDRNVVIYDNSNNAIWATHTNVGNAEITVIPHSNGTAAASGAAQNKVNELYISMY
XP_020579629.1	*Phalaenopsis equestris*	MHSAKQSIITQTPPKSQLNHKTMASSISTAAFLLLLVGTLLSLQTAPVSAADRLNAGQSLGGGQSLAQGPYIFIMQKDCNLVLYDNNKAVWATGTNGKA<br>SGCYVTMQRDGNLVIYSGTRVIWASNTNRQNGNYYLILQRDRNVVIYDNSNNAIWATGTNVGNAAIVVIPHSNGTAAASGAAQNKVKELYP
XP_020574364.1	*Phalaenopsis equestris*	MVFRDPNLHYHALSQAKNYNTHSPQVPNKTLLSLQTAPVSAADRLNAGQSLGGGQSLAQGPYIFIMQKDCNLVLYDNNKAVWATGTNGKASGCYVT<br>MQRDGNLVIYSGTRVIWASNTNRQNGNYYLILQRDRNVVIYDNSNNAIWATGTNVGNAAIVVIPHSNGTAAASGAAQNKVKELYP
XP_020579587.1	*Phalaenopsis equestris*	MTFFITFPSIFLLSLAVLSILPTTPASAASTLYAGQSLNPGQSLTQGNYSFIMQQDCNLVLYDKNRAVWSTETSGRASGCVLRLQINGNLIIYSGVRVIWQS<br>NSSGFVGIYYLILQTDRNVVIYDFFHDAIWSTGTEVPSADSVDDKAAPFAVAKEKVVEIDNVLEG
XP_020598103.1	*Phalaenopsis equestris*	MVFSSMIKSLLFAASLTILLANPSSAQNYNHLLAGQRLNTGESLRTDNYIFIIQYDCNLVLYESNTAIWASGTDGRGQGCYLTMQHDGNLVVYDYTNTA<br>VWASNTDRENGNYILIVQRDRNVVIYSNPIWATGTNYAGSVGVVVAAARNGTVGVSGAKQNKVREMGNIREVIN
XP_020598757.1	*Phalaenopsis equestris*	MVFSSMIKSLLFSASLTILLANPSSAQNYNHLLAGQRLNTGESLRTDNLKFIIQYDCNLVLYVNNIAIWASGTNGKGQDCYVTLQHDGNLVVYDYINTAV<br>WASKTDRENGNYILILQRDRNVAIYSNPIWSTETNYAGSVGVVVAAAHNGTVGGSGAKQNKVREMGKIMEVIN

XP_020595050.1	*Phalaenopsis equestris*	MAFFPMIKTLILFSASLTILLAIPSSAQTYNHLLAGERLNPGDSLVQGHYFFTMQYDCNLVLYDYSTPLWASGTQNKGSGCYAIMQRDGNLVVYDSNNN<br>PLWASNTNGEEGNYILILQKDRNVVIYSNPIWATGTNQVGWIGVVVAAARNGTVGVSGAEQNKVREMGKIMEV
XP_020572028.1	*Phalaenopsis equestris*	MASYPMFKTLILCAAFLTILLANPSSAQYYNHLLAGERLRPGESLVQGHYFFTMQYDCNLVLYDDSTPIWASGTQGKGSGCYVIMQQDGNLVIYDSKKK<br>PLWASNTNVGKGNYVLVLQKDRNVVIYSKPIWATGTNKVGWTGVVVAAAAGNGTVGVSGAEQNKVREMRKFVELMNDV
XP_020576869.1	*Phalaenopsis equestris*	MVLSQMIKSLLFAASLTILLADPSSAQTYNHLLSGERLNGGESLRSMNLQLIIQYDCNLVLYDSNSAIWASGTGGSGSGCYLAMQNDGNLVIYDYSNRAI<br>WASNTGRDNGYYILVLQKDRNVVIYGNPIWATGTNYGGSVAVVVTAARNGTVGVSAAKQNKVRKMGKIMEGMI
XP_020597564.1	*Phalaenopsis equestris*	MVLSPMIKSLLFAAALTILLAKPSSAQTDNYLLSGERLEGGKSLKSGNLEFIIQDDCNLVLYHSKKAVWASGTDGFSLSCYLVLQQDGNLVIYNYLDRAI<br>WESYTFGIDGHYILVLQKDRNVAIYGDRIWATENKDLGSMAVVVAAARDGTVGVSGAKQNMVRKMGKIMEVIN
XP_020576867.1	*Phalaenopsis equestris*	MVLSPMIKSLLFAAALTILLAKPSSAQTDNYLLSGERLEGGKSLKSGNLEFIIQDDCNLVLYHSKKAVWASGTDGFSLSCYLVLQQDGNLVIYNYLDRAI<br>WESYTFGIDGHYILVLQKDRNVAIYGDRIWDTDNKYPGSVAVVVAAARDGTVGVSGAKQNMARKMGKIMEVIN
XP_020676062.1	*Dendrobium catenatum*	MASSPSSPTILLLLLSVATLFSLLTTPASAANRLNAGQSLGPGQSLAQGPYLFIMQPDCNLVLYDNIKAVWATGTNGRASGCVLKMQTDGNLVIYSGSRV<br>IWASNTNRQNGNYYLLLQRDRNVVIYDNSNNAIWATGTNVGNAAVVVIPHSNGTAAASGAAQNKVKELYP
XP_020688140.1	*Dendrobium catenatum*	MASSPSSPAILLLFLTVATLFSLLTTPASAANRLNAGQSLGPGQSLAQGPYLFIMQQDCNLVLYDNNRAVWATGTNGRAFGCVLKMQTDGNLVIYSGSK<br>VIWASNTNRQIGNYYLLLQRDRNVVIYDGANNAIWATGTNIGNAAVVVIPHSNGTAAASGAAQNKMKELYP
XP_020695144.1	*Dendrobium catenatum*	MASSPSSPTILLLLLSVATLFSLLITPASAANRLNAGQSLGPGQSLAQGAYLFIMQQDCNLVLYDNNRAVWATGTNGRASGCVLKMQTDGNLVIYSGSR<br>VIWASNTNRQNGNYYLLLQRDRNVVIYDNSNNAIWATGTNVGNAAVVVIPHSNGTAAASGAAQNKVKELYP
XP_020692119.1	*Dendrobium catenatum*	MASSPSSPTILLFLLLSAATLFSLLTTPASATNRLNAGQSLGPGQSLTQGPYLFIMQPDCNLVLYDNIKAVWATGTNGRAFSCVLKMQTDGNLVIYSGSRV<br>IWASNTNRQIGNYYLLLQRDRNVVIYDSANNAIWATGTNVGNAAVVIIPHSNGTAAASGAAQNKMKELHP
XP_020676077.1	*Dendrobium catenatum*	MASSPSSPTILLLFVATLFSLLTTPAFADDRLNAGQSLGPGQSLAQGGYLFIMQQDCNLVLYDNGGAVWATGTNGRASGCVLRMQTDGNLVIYSGSNVI<br>WASNTNRQNGNYYLIVQRDRNVVIYDNSNNAIWATGTNVGNAVVVVIPHSNGTAAASGAAQNKVKKLHP
XP_020702983.1	*Dendrobium catenatum*	MASSPTILLLLLLSATALFSLLTPPVSADDRLNAGQSLNPGQSLAEGTYLFIMQQDCNLVLYDNGGAVWASGTNGQASDCVLTMQTDGNLVIYSNGDAI<br>WASNTNRQDGDYYLILQRDRNVVIYDNANNAIWATGTNVGNADVFVIPHSNGTAATSGAAQNKVKELYP
PKU68029.1	*Dendrobium catenatum*	MASFTFFLFAATLTVLLATPASCQYYNHLLAGERLNTGQALTQGGYSFIIQSDCNLVLYEYGSAIWSSGTNGKGKGCYVTMQTDGNLVIYNRKNKAIWA<br>SNTNRETGNYILILQKDRNVVIYSLPIWATGTNTVGSAGVAIAGAGNGTVAVSGAEQNKVREMGKMVELKSDE
XP_028555350.1	*Dendrobium catenatum*	MAFLIRTLLLCAASLTFLAAPSSGQLFNHLLDGERLGTGQALTQGGFAFVIQSDCNLVLYEFGNPLWSSGTNGQGLGCYVTLQSDGNLVIYDQSNKAIW<br>ASNTNGETGNYLLILQKDRNVVIYSLPIWATGTNTVGSAGVVIAGARNGTVGVTGAEQNKVREMGKIMEECCPNDINGAYAADRKFETLKTKSFRRSNP<br>NPLIQIKSAKQPQQSAMASFTFFLFAATLTVLLATPASCQYYNHLLAGERLNTGQALTQGGYSFIIQSDCNLVLYEYGSAIWSSGTNGKGKGCYVTMQTD<br>GNLVIYNRKNKAIWASNTNRETGNYILILQKDRNVVIYSLPIWATGTNTVGSAGVAIAGAGNGTVAVSGAEQNKVREMGKMVELKSDE
PKU68030.1	*Dendrobium catenatum*	MAFLIRTLLLCAASLTFLAAPSSGQLFNHLLDGERLGTGQALTQGGFAFVIQSDCNLVLYEFGNPLWSSGTNGQGLGCYVTLQSDGNLVIYDQSNKAIW<br>ASNTNGETGNYLLILQKDRNVVIYSLPIWATGTNTVGSAGVVIAGARNGTVGVTGAEQNKVREMGKIMEVEGDE
XP_020702049.1	*Dendrobium catenatum*	MAFFSMIKILLLCAASLSVLLLATPASGQSYNHLLSGERLNAGQSLIQGSYQFIIQNDCNLVLYMYGTPRWASNTGGQASGCYLAMQSDGNLVVYDYGN<br>RAIWASNTGGENGYYNLILQKDGNVVIYGKPIWATGTSYSGSAVVVVADARNGTVGASGAEQNKVKEMGKIVQVHGDE
XP_020685020.1	*Dendrobium catenatum*	MTFSISSAMIFLLSLALFSTLVSADNHLLPGDRLNPGNFLKQDRYMLIMQEDCNLVLYNLNKPEWATKTANRGSRCFVTLQSDGNFVIYDDHEERNEAIW<br>ASNTDGQNGNYVIILQKDGNLVLYSKPIFATGTNRFGSTAVVVAKRNRKAHFGVEQNIIEVTTNL

PKU80952.1	*Dendrobium catenatum*	MMASTTISICVAILLLLIFAPSNSSCRTTMNSGDTLITSDSLVLGFCGFTMQSDCNLVLYVLGRSVWDTKSYGRGDRCQLELTRNGQLVISNNGVSVWKN<br>SFQGEDDNYVLKLQEDGNVVIFNSNENGVWFTNTSVSCS
XP_028552382.1	*Dendrobium catenatum*	MAMLRQASFFYFLALFMAACGLFAGGSMAQMSHMLSGDVLKTGQNISNPLYTLVMQADCNLALYRSPSVFVWSTGTNGKGSGCELWLKTDGNLVIN<br>DKDEKLIWQTGTNGTVGHYVLLLQRDRNLVIYSVPAWDSGTGTRPKLKIKYSNIEASASSTGVSTAPTNQLLPGDILKTGQKISNANYTLLMQPDCNLVL<br>YGSSSQAIWYTHTNDKGSECELRLQTDGNLAIHDKDGKLIWQTATNGTIGNYVLLMQRDHNVVLYGKPIWDSGTGSVRVPPTTY
XP_020688646.1	*Dendrobium catenatum*	MAFSISSTMIFLLSIALFSTLVSADNHLLPGERLNPGNFLKQDRYMLIMQEDCNLVLYNLNKPEWATNTANQGSRCFVTLQSDGNFVIYDEQEGRNEAIW<br>ASKTDGENGNYVIILQKDGNLVLYSKPIFATGTNRFGSTAVVVAKRNRKAHFGVEQNIIEVTTNL
XP_020702047.1	*Dendrobium catenatum*	MIKVLLLCTTSLSLLFTTPGSGQYSNYLLSGERLDTDRYLTKMVYKFIMQNDCNLVLYQLTNLIWSSGSGFYLTFERDGNLIIHNNENEIIWETEAIRSEGK<br>YILILQRGGIVAAYGPSVWSTETNGYGSDEVVVAIALNGTMGVSGGEQNNVRETGKIMEVI
XP_020682802.1	*Dendrobium catenatum*	MTSMKNSPSSAAKSSILLSLAVLLLLNAPASCSSESFNNVLSSGYPLNAGCSLVQGKYNFTMQYDCNLVLYESEVAIWSSQTDGKGSNCSLVLQNYGELF<br>ISTAAGIIVWRSETGGEYGHFVLVLQPNGDVVVYGDAVWSTGTYRAPIAISGDKP
PKU64224.1	*Dendrobium catenatum*	MQKDCNLVLYDNQTALWSSHTDNKGVNCFLVLQNNGELVIISNYRITVWRSETGGQAGKYALVLQPNGDVVVYGNPVWSTGTSSATFLVSIAVLVILA<br>TSTDHSAKFYGNVLKSGGKLDTGESLVHGNYSFVMQKDCNLVLYDNQTALWSSHTDNKGVNCFLVLQNNGELVIISNYRITVWRSETGGQAGKYALV<br>LQPNGDVVVYGNPVWSTGTSNIEIPKH
XP_020703015.1	*Dendrobium catenatum*	MTITTYSAKSSVALLSTTLLLLLISSSSSTSFSTNINVLKSGQRLHSGSFLSLGNYVFAMQADCNLVLYNNNTILWSSNTGGKGKNCYLSLESDGELHIYGD<br>GGRKVWRSETGGEYGDHALVLQPNGNVVIYGSPVWSTGLSYQTPNTDNYEQNTIN
KAI0524813.1	*Dendrobium nobile*	MASSPSSPTILLLLLTVATLFSLLTTPVSAANRLNAGQSLGPGQSLAQGPYLFIMQQDCNLVLYDNNRAVWATGTNGRAFGCVLKMQIDGNLVIYSGSK<br>VIWASNTNRQIGNYYLLLQRDRNVVIYDSANNAIWATGTNIGNAAVAVIPHSNGTAAESGAAQNKVNELYP
KAI0499027.1	*Dendrobium nobile*	MASSPSSPTILLLLLSATALFSLLTTPASAANRLNAGQSLGPGQSLAQGPYLFIMQPDCNLVLYDNIRAVWATGTNGRAFGCVLKMQTDGNLVIYSGSRVI<br>WASNTSRQIGNYYLLLQRDRNVVIYDSANNAIWATGTNVGNTAVVVIPHSNGTAAASGAAQNKVKELHP
KAI0519187.1	*Dendrobium nobile*	MASSFSSPTILLLLLSAATLFGLLTTPASAANRLNAGQSLGPGQSLAQGAYLFIMQQDCNLVLYDNNKAVWATGTNGRASGCVLKMQADGNLVIYSGSR<br>VIWASNTNRQNGNYYLILQRDRNVVIYDNSNNAIWATGTNVGNAAVVVIPHSNGTAAHPARRRTR
KAI0519205.1	*Dendrobium nobile*	MASSLSSPTILLLLSTAALFSLLTTPASADNRLNANQLLEPGQSLAQGAYLFIMQQDCNLVLYDNGGAVWATGTNGQASDCVLNMQNDGNLVIYSDSN<br>VIWASNTNRQDGDYFLLLQRDRNVVIYDNANNAIWATGTNIGNAAVDVIPHSNSTAATTGAAQNKVKELYP
KAI0519194.1	*Dendrobium nobile*	MASSPTILLLLLFATALISLLTPPVSADDRLNAGQSLNPGQSLAEGAYLFIMQQDCNLVLYDNGGAVWSSGTNGQASDCILTMQTDGNLVIYSNGDAIWA<br>SNTNRQDGDYYLILQRDRNVVIYDNANNAIWATGTNVGNAYVFVIPHSNDTAAMSGAAQNKVKELYP
KAI0519204.1	*Dendrobium nobile*	MASFPSSPTILLLLSAAALFSLLTPPVSADDRLNSGQSLNTGQSLTQGAYVFIMQLDCNLVLYDNGHAVWASGTDEKASDCVLTMQSDGNLVIYSNGDAI<br>WASKTNREDGYYFLILQSDRNVVIYDNSNNPIWATGTNVVNADVFVIPHSNGTAAASGTAQNKVKLYP
KAI0519202.1	*Dendrobium nobile*	MAFPPSSPTILPLLLSTAALCSLLTPSVSADDRLDAGKSLNTGQSLTQGDYVFIMQQDCNLVLYDNGHAVWATGTDEQASDCVLDMQTNGNLVIYGNG<br>VVIWGSDSNRQEGYYFLILQTDRNVVIYDSSNNPIWATGTNVVNADVFVFPHSNGTAAASGAAQNKVKELYP
KAI0519203.1	*Dendrobium nobile*	MAFPPSSPTILLLLLSTAALCSLLTPSVSADDRLDAGKSLNTGQSLTQGAYVFIMQQDCNLVLYDNGHAVWATGTDEQASDCVLDMQTNGNLVIYGNG<br>VVIWGSDSNRQEGYYFLILQTDRNVVIYDSSNNPIWATGTNVVNADVFVFPHSNGTAAASGAAQNKVKELYP
KAI0511066.1	*Dendrobium nobile*	MASFTFFLFAATLTVLLATPASSQYYNHLLAGERLNTGQALTQGGYAFIIQSDCNLVLYEYGSAIWSSGTNGKGKGCYVTMQTDGNLVIYNRKNKAIWA<br>SNTNRDTGNYILILQKDRNVVIYSLPIWATGTNTVGSAGVAIAGAGNGTVGVSGAEQNKVREMGKMVEIKSDE

KAI0519125.1	*Dendrobium nobile*	MAFFIKTLLLCFASLTLLAAPSSGQLFNHLLDGERLGTGQALTQGGFAFVIQSDCNLVLYEFGNPLWASGTNGQGLGCYVTLQSDGNLVIYDHSNKAIWASNTNGETGNYLLILQKDRNVVIYSLPIWATGTNTVGSAGVVIAGARNGTVGVTGAEQNKVREMGKIVEVEGDE
KAI0519123.1	*Dendrobium nobile*	MAFFSLIKILLLCTASLSVLLLATPVSGQNYNHLLSGERLNQGESLTQGNYQFIIQNDCNLVLYIYGTPRWASNTGGLASGCYLAMQSDGNLVVYDYGNRAIWASNTGGENGYYNLILQKDGNVVIYGKPIWATGTSYSGSAVVVVAAARNGTVGASGAEQNKVKEMGKIVQVHGDE
KAI0519122.1	*Dendrobium nobile*	MAFFSMMKIFLLCTASLSLLLATPASGQNHLLPGERLNSGQSLIEGSYTLTIQRDCNFVLYKDGEPKWASQTGHRQGSICYVTMQTDGNLVIYGYENRAIWASNTAGEKGNFVFLLQKDGNAVIYSKPTWSTGTSSYGSASVVIKPGLNGTVGAYGAEKNKVRKMGKIMEVMRDE
KAI0512221.1	*Dendrobium nobile*	MAINLASPRTSAISLLSSSILLLLISSGSSTSLGNVLKNGYELGAGGLLSIGNYKFIMQRDCNLVLYENNTVLWETKTQGKGTLCSLSLQSNGELFVFSESRKALWRSETGGEFGNYALVLQPNGNVVVYGSPMWSTGTYIQSTASNAPPRYFVSPNP
KAI0488594.1	*Dendrobium nobile*	MAFSISSTMIFLLSIALFSTLVSADNHLLPGERLNPGNFLKQDRYMLIMQEDCNLVLYNLNKPEWATKTANQGSRCFATLQSDGNFVIYDEQEGRNEAIWASKTDGENGNYVIILQKDGNLVLYSKPIFATGTNRFGSTAVVVAKRNRKAHFGVEQNIIEVTTNL
KAI0519118.1	*Dendrobium nobile*	MAFFMIKVLLLCTTSLSLLTTPASGQQYNYLLSGDRLNTGKALIQGNYKFIIQDDCNLVLYQFREPVWSSKTSGLDSDCYLTLNETGILIINNNANHHIWTSNTANGKNGNYILILKNSHGAAVYGPSIWSTELKPKGSNDVVIATALNGTTGVSGEEQNKVREMGKIMEVI
KAI0512280.1	*Dendrobium nobile*	MTSMKNTPSSTAKSSILLSLAVLLLLNAPASCSSESFNNVLSSGYPLNAGCSLVQGKYSFTMQYDCNLVLYESEVAIWSSQTDGKGSNCSLVLQNNGELFISPAAGIIVWRSETGGEYGHFVLVLQPNGDVVVYGDAVWSTGTYRAPHAISGDKP
KAI0512223.1	*Dendrobium nobile*	MAINIVSASTLLSSSIFLLLIASSSSTRFGYPASPGNVLRSGEELSTDKSLSIGNYTLIMQKDCNLVLYENDKAIWESSTSEMGINCSLVLQSNGELHIFSDVGPPVWRTETSQRDGKYALVLLPNGNVVVYGSPIWSTGTHTQRKAMNAPPTNYAPKIENP
KAH0465276.1	*Dendrobium chrysotoxum*	MASSLSSPTIFLLLLSAATLFSLLTTPASAANRLNAGQSLGPGQSLAQGPYLFIMQQDCNLVLYDNNKAVWATGTNGRASGCVLKMQIDGNLVIYSGTRVIWASNTNRQIGFYYLILQRDRNVVIYDSSNNAIWATGTNIGNAAVVVIPHSNGTAAASGATQNKVKELYP
KAH0465907.1	*Dendrobium chrysotoxum*	MASSLSSPTILLLLLSAATLFSLLTTPASAANRLNAGQFLGPGQSLAQGPYLFIMQQDCNLVLYDNNKAVWATGTNGRASGCILKMQTDGNLVIYSGTRVIWASNTNRQIGFYYLILQRDRNVVIYDSSNNAIWATGTNIGNAAVDVIPHSNGTAAASGAAQNKVKELYP
KAH0465275.1	*Dendrobium chrysotoxum*	MASSPSSPTILLLLSAAALFSLLTTPASARDRLTAGEFLEPGQSLRQGAYSFIMQHDCNLVLYDNGRAVWATGTNGQASGCELRMQNDGNLVIYSGRRAIWASKTNRQMNFYYVILQRDRNVVIYSIGGYAIWATGTNVGNAAVVVIPHSNGTAAASGAAQNKDLSTQMAGKLIFSLDGNLIVEGINYGNFIQFEMQFEDFKLSVDGEEMIDLSAGIRRNVPLVKITLGSGVDSPIFKMEDDVTPLPIVPFA
KAH0464855.1	*Dendrobium chrysotoxum*	MIKVLLLCAASLSVLLLATPASGQSYNHLLSGERLNAGQSLIQGSAQLIIQDDCNLVLYIYGAARWASSTGGLASGCYLAMQTDGNLVVYDYGNRAIWASNTGRENGNYILILQKDYNVVIYGNPIWATETSYSGSAVVVIAAARNGTVGASGAEQNKVKEMGKIVQVVQGDA
KAH0465791.1	*Dendrobium chrysotoxum*	MAFFSTNKILLLCAASLSVLLLATPASGQSYNHLLSGERLNAGQSLIQGNYQFIIQNDCNLVLYIYGSPRWASNTGGLASGCYLAMQTDGNLVVYDYGNRAIWASNTGGQNGYYNLILQKDGNVVIYGKPIWATGTSYSGSAVVVIAAARNGTVGASGAEQNKVKEMGKIVQVVQGDE
KAH0465460.1	*Dendrobium chrysotoxum*	MQTDGNLVIYGYENRAIWASNTAREKGDFVFLLQKDGNGVIYSQPIWSTGTTYYGSASVVIKAALNGTVGASGAKQNKVLLLCTTSLSLLLTTPASGNNYLLSSERLNTGYSLIEGVYQFIIQNDCNLVLYQFRDPIWSSGTNNQGSGCYLTFEKDGNLVIYDYENKIVWETKTNGIEGNYVLILQRDRNVVIYGPGGWATKTNAYGSNNVVIATALNSTMGVSGEEQNKVLLLCTTSLSLLLTTPASGSNYLLSSERLNTGYSLIEGVYQFIIQNDCNLVLYHFRDPIWSSGTNNQGSGCYLTFEKDGNLVIYDYENKIVWETKTNGIEGNYVLILQRDRNVVIYGPGRWATKTNAYGSNNVVVATALNGTMGVSGEEQNKVMKMGKIMEVMRDE
KAH0448068.1	*Dendrobium chrysotoxum*	MAFSIRSETVFVLSVVIFSTLAAAENYLLPGDWLNRGASLQEGKYTFTLQEDCNLVLYNSKKPVWASETANKSTYCFAILQPDGNFVITESIFSLTEPIWASNTGGKNGNYVLILQRDGNVVLYSNPIFSTGTNKIGSADVVASLKSNRKVLPGAEQNKVFEMKKVIVDVAGSL

KAH0464854.1	*Dendrobium chrysotoxum*	MAFFSMIKVLLLCTTSLSLLATLALGQNSTNYLLSGERLNTNQYLIEGNTKFIIQNDCDLVLYKSQVLVWHSNTTNEGSGCYLIFQPNGKLVIFDNKNEVV<br>WDTTSIREIGNYILTLNRDHNVVIYGPKVWDNENNTIGSDDVVVATSLNGPMGASGVKNNKMMEMGKIIEVMSNE
KAH0466093.1	*Dendrobium chrysotoxum*	MAFFSMIKVLLLCTTSLSLLATLALGQNSTNYLLSGERLNTNQYLIEGNTKFIIQNDCDLVLYKSQERAWHSNTTNEGSGCYLIFQPNGKLVIFDNQNEVV<br>WDTTLTREIGNYILTFNRDHNVVIYGPKVWANENNTIGLDDVVVATSLNGPMGASGVKNNKMMEMVKIIEVMSNE
KAH0465987.1	*Dendrobium chrysotoxum*	MAFFSMIKVLLLCTTSLSLLVTLALGQNSTNYLLSGERLNTNQYLIEGNTKFIIQNDCDLVLYKSQVLVWHSNTTNEGSGCYLIFQPNGKLVIFDNKNEVV<br>WDTTSIREIGNYILTLNRGHNVVIYGPKVWDNENNTIGSDDVVVATSLNGPMGALGLKNNKMMEMGKIIEFMSNE
KAH0461948.1	*Dendrobium chrysotoxum*	MTITTYTAKSSVALLSTTILLLLISSSSSTSFFSNVNVLKSGQRLDSGSFLSYGNYAFAMQADCNLVLYNNNTVLWSSHTDGKGKDCYLSLVPDGELYIYG<br>DGGKMVWRSETGGEYGHYALVLQPNGNVVIYGSPVWSTGLSYQTPNAYQTPNPGNYEANTFKSNNP

Supplementary Table S2. Sequences of conserved motifs in GAFPs

Motif	Sequence
Motif1	LAQGNYLFIMQNDCNLVLYDNNKAVWASGTNGKASGCYLKMQTDGNLVIY
Motif2	SGNRAIWASNTNRZBGNYYLILQRDRNVVIY
Motif3	NAAVVVIPHSNGTAAASGAAQNKVKELYK
Motif4	TTPASAANRLLAGZRLNTGQS
Motif5	NNPIWATGTNV
Motif6	MASSPSSPTJLLLAL
Motif7	FGSTAVVVAKRNRKAHFGVEQNIIEVTTN
Motif8	TLFSLL
